# Small and Long Regulatory RNAs in the Immune System and Immune Diseases

**DOI:** 10.3389/fimmu.2014.00513

**Published:** 2014-10-20

**Authors:** Anna Stachurska, Maria M. Zorro, Marijke R. van der Sijde, Sebo Withoff

**Affiliations:** ^1^Department of Genetics, University Medical Center Groningen, University of Groningen, Groningen, Netherlands

**Keywords:** regulatory RNAs, miRNAs, long non-coding RNAs, immune system, autoimmune diseases

## Abstract

Cellular differentiation is regulated on the level of gene expression, and it is known that dysregulation of gene expression can lead to deficiencies in differentiation that contribute to a variety of diseases, particularly of the immune system. Until recently, it was thought that the dysregulation was governed by changes in the binding or activity of a class of proteins called transcription factors. However, the discovery of micro-RNAs and recent descriptions of long non-coding RNAs (lncRNAs) have given enormous momentum to a whole new field of biology: the regulatory RNAs. In this review, we describe these two classes of regulatory RNAs and summarize what is known about how they regulate aspects of the adaptive and innate immune systems. Finally, we describe what is known about the involvement of micro-RNAs and lncRNAs in three different autoimmune diseases (celiac disease, inflammatory bowel disease, and multiple sclerosis).

## Introduction

**Figure 1:**
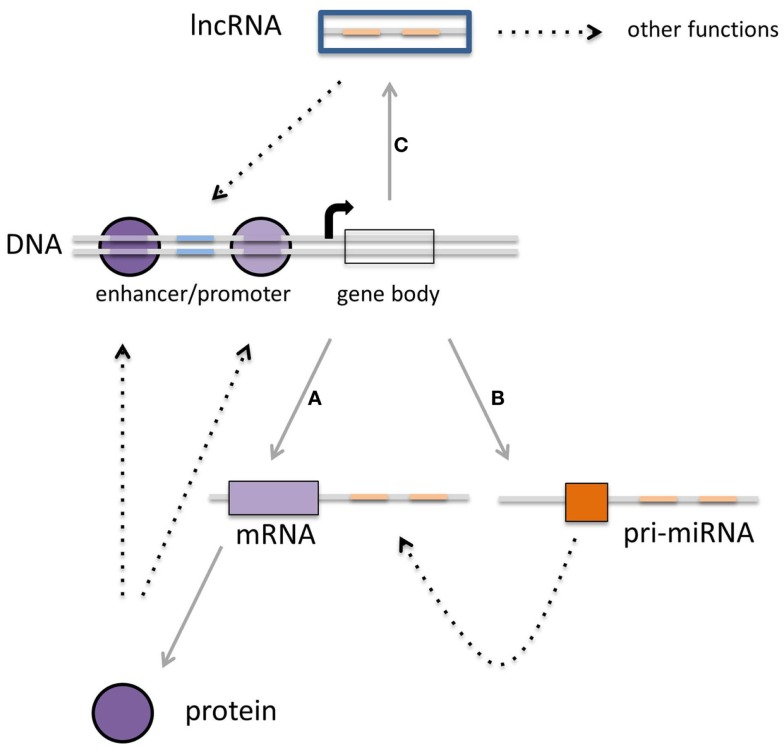
**Multiple layers of gene expression controlled by transcription factors, miRNAs, and lncRNAs**. **(A)** Protein-coding genes are transcribed into mRNA, which subsequently are translated into proteins. These proteins can function as the classical transcription factors. **(B)** There is a second class of RNAs that is not translated into protein but rather is regulating the expression of other transcripts. The third class of transcripts described in this review **(C)** is the long non-coding RNAs that can regulate gene expression as well, although other functions for these transcripts have been described (see Figure 3). It is becoming clear that there is interaction within each class, but also between these three classes, which can converge on transcriptional outcome (see text for details).

The discovery of the first micro-RNA (miRNA) in 1993 (1, 2) was the start of research that has led to the understanding that gene regulation is not only controlled by proteins (transcription factors) but also RNA molecules. Since then, thousands of novel non-coding RNAs, which can be subdivided into dozens of families (3), have been identified. Two of the most widely studied classes of non-coding RNAs, miRNAs and long non-coding RNAs (lncRNAs), are now recognized as important regulators of gene expression. These molecules are also designated as (small or long) regulatory RNAs. At the time of writing this review, the authorative miRNA database miRBase (release 21) describes 1,881 human miRNA precursors and 2,588 human mature miRNA sequences (4), whereas the GENCODE compendium (V19) mentions 13,870 human lncRNA genes (5). MiRNAs are thought to affect gene expression by inhibiting target mRNA translation (which leads indirectly to degradation of the target) or they can directly induce target mRNA degradation. Many lncRNAs are thought to be involved in chromatin modification processes that, in turn, affect gene expression levels (Figure 1). The role of miRNAs in homeostasis and the deregulation of miRNAs in human disease have been well established, but the role of lncRNAs in these processes is not yet fully appreciated. Here, we will review what is known about the role of miRNAs and lncRNAs in the development and activation of the adaptive and innate immune systems in health and disease.

## MiRNAs

Micro-RNAs are short (19–24 nt), single-stranded, RNAs that are involved in the post-transcriptional regulation of gene expression. Their sequences are evolutionary strongly conserved. miRNA expression profiles and target mRNA sites are also conserved, allowing the translation of findings in mouse models to human physiology.

**Figure 2:**
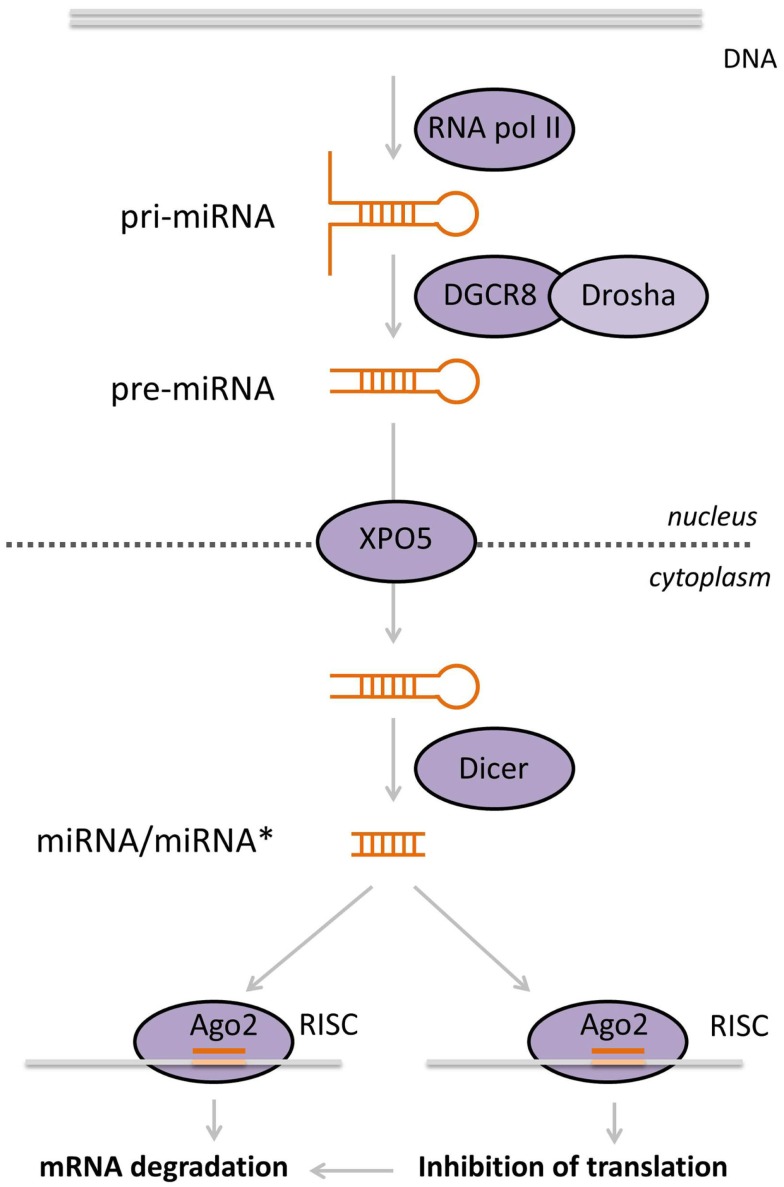
**Biogenesis of miRNAs**. MiRNAs are transcribed by RNA pol II in the nucleus. Double-stranded miRNA hairpins in the pri-miRNA transcript are recognized and cleaved by the microprocessor complex, composed of Drosha and DGCR8, producing the pre-miRNA. These are subsequently exported into the cytoplasm by XPO5, where they are recognized by Dicer, which cuts off the loop of the hairpin yielding a small double-stranded RNA molecule. One of these strands, the mature miRNA, is loaded into the RISC complex that contains AGO2. This complex is guided to the target mRNA, based on sequence homology between the miRNA and the target. Ultimately, this leads to diminished mRNA translation and/or degradation of the target.

Micro-RNAs are transcribed by RNA polymerase II into longer (several hundred to several thousand nucleotides) primary miRNAs (pri-miRNAs), containing a cap as well as a poly-A tail. The pri-miRNA is processed in the nucleus by a *microprocessor*, a complex composed of Drosha (a class III RNase) and the DiGeorge syndrome critical region gene 8 (DGCR8), into a ~60 nt precursor-miRNA (pre-miRNA). In this step, the double-stranded stem-loop structures are specifically recognized by the microprocessor, which catalyzes the cleavage of the pri-miRNA near the base of the stem (6, 7). Then, the double-stranded pre-miRNA stem-loop structure is transported into the cytoplasm by a complex containing exportin 5 (XPO5), where it is recognized and further processed by a class III RNase named Dicer into a double-stranded RNA duplex of ~19–24 nt in length. Next, only one of the strands is incorporated into the RNA-induced silencing complex (RISC) composed of Argonaute (AGO) and GW182. The RISC complex is guided to the 3′-untranslated region (3′-UTR) of target mRNA molecules. This leads successively to a decrease in target stability, resulting in accelerated uncapping and deadenylation (8) and/or inhibition of translation (9) (Figure 2). It has been suggested that the translational repression of mRNAs takes place in specialized compartments called processing bodies (P-bodies), compartments in the cytoplasm involved in the storage, and degradation of repressed mRNAs (10). To make things more complex, miRNAs were shown to be transported to the nucleus, where they can affect their own expression or the expression of other miRNAs (11). Moreover, lncRNAs can act as sponges for miRNAs. It was demonstrated that the lncRNA phosphatase and tensin homolog *(PTEN)* pseudogene1 (*PTENpg1*) sequesters various *PTEN*-targeting miRNAs, thereby indirectly regulating the PTEN mRNA level (12).

Reports describing that miRNAs can originate from other regulatory RNAs, like tRNAs (13) or pre-ribosomal RNAs (14), complicate the “canonical pathway of miRNA production.” MiRNAs can also be derived from introns, which mimic the structural features of pre-miRNAs and these miRNAs can, therefore, enter the miRNA processing pathway independent of Drosha (15). This group of miRNAs can even be subdivided into two groups: (a) splicing-dependent miRNAs (mirtrons, e.g., human *miR-877* and *miR-1226*) or (b) splicing-independent simtrons (human *miR-1225* and *miR-1228*). The processing of mirtrons requires the spliceosome but not Drosha or DGCR8, whereas the generation of simtrons depends on Drosha (but not on DGCR8). Mirtrons are exported from the nucleus by XPO5, cleaved by Dicer, and subsequently enter the RISC complex, similarly to canonical miRNAs. We do not yet know what factors regulate simtron export from the nucleus, but simtrons also enter the RISC complex in the cytoplasm (16).

Interest in miRNAs grew when it was found that miRNAs can be detected in many body fluids such as serum, cerebrospinal fluid (CSF), saliva, and urine (17) and that miRNA profiles are remarkably stable (e.g., resistant to RNases, freeze-thaw cycles). This protection from degradation is probably conferred by one or more mechanisms: (1) miRNAs can be bound to protein components of the RISC complex (AGO2), (2) they can be bound to high density lipoproteins, or (3) they can be packaged in exosomes (18). It was exciting to discover that miRNA profiles in circulation can be disease or even disease stage specific (19). Moreover, they can be useful in predicting treatment response (20). Research into the biological role of circulating miRNAs is still in its infancy, but recent papers describe the intriguing possibility that miRNA can be secreted by one cell type and can then exert its function on or in other cell types (21–23). Exosomes have been shown to participate in various processes that are crucial for immune system function and they can be released by various immune cell types, e.g., T-cells, B-cells, and dendritic cells (DCs). Importantly, these exosomes contain miRNAs, some of which are cell-type specific, while others are present in exosomes of various cell types. Moreover, some miRNAs are more highly expressed in exosomes than in the cells that excrete them, implying that a subset of miRNAs is specifically packaged (24). The selection of miRNA for packaging into exosomes has been described based on two specific motifs in the miRNA sequence; these are recognized by sumoylated heterogeneous nuclear ribonucleoprotein A2/B1 (hnRNPA2B1), a protein controlling miRNA loading into exosomes (25).

## Long Non-Coding RNAs

Long non-coding RNAs are a heterogeneous group of non-coding transcripts longer than 200 nucleotides (26, 27) and they constitute the major class of regulatory RNA genes (28, 29). Thousands of mammalian lncRNAs have been identified since the first genome-wide discovery studies in the early 2000s and it has become clear that they play important roles in regulating several biological processes, such as gene expression, chromatin remodeling, and protein transport. Although many lncRNAs have been identified, little is known about either their general characteristics or their possible mechanisms of action in health and disease. They can be detected both in the nucleus and in the cytosol, and can be polyadenylated or not. Compared to protein-coding genes, lncRNAs have fewer but longer exons, which are poorly conserved across species (26, 30). In general, the expression of lncRNAs is lower than that of protein-coding genes, although in a cell-type-specific context the expression can be just as high (26). There is growing evidence pointing to changes in lncRNA expression being associated with the etiopathology of diseases, for instance in cancer and autoimmune disease (18, 31). Expression profiling of specific immune cell subsets has revealed an enrichment of long intergenic non-coding RNAs (lincRNAs) that are expressed in immune cells in autoimmune disease-associated loci, thereby implying that these non-coding RNAs play a role in the etiology of autoimmune disease (Barbara Hrdlickova, personal communication). Furthermore, expression quantitative trait locus (eQTL) analysis has demonstrated that disease-associated single nucleotide polymorphisms (SNPs) can affect the expression of lncRNAs, relating lncRNAs to disease susceptibility (32).

Long non-coding RNAs are a structurally and functionally heterogeneous group of transcripts. One approach classifies them into four different subclasses based on their location with respect to the closest protein-coding gene (5, 18). The largest subclass consists of the lincRNAs, which do not overlap with protein-coding genes. Of the remaining “genic” lncRNAs (the second largest subclass), the antisense lncRNA group contains transcripts that overlap with exons of protein-coding genes on the opposite strand (natural antisense transcripts, or NATs) or transcripts that reside in an intron of the protein-coding gene on the opposite strand (antisense intronic transcripts). The antisense and sense transcripts are often co-expressed. The third subclass of lncRNAs encompasses the sense lncRNAs. These transcripts can contain coding genes within an intron on the same strand (sense overlapping transcripts), or they can be located within an intron of a protein-coding gene on the same strand (sense intronic transcripts). The fourth subclass comprises the bi-directional or divergent lncRNA transcripts. These are antisense transcripts that co-transcribe in the opposite direction to the protein-coding gene.

**Figure 3:**
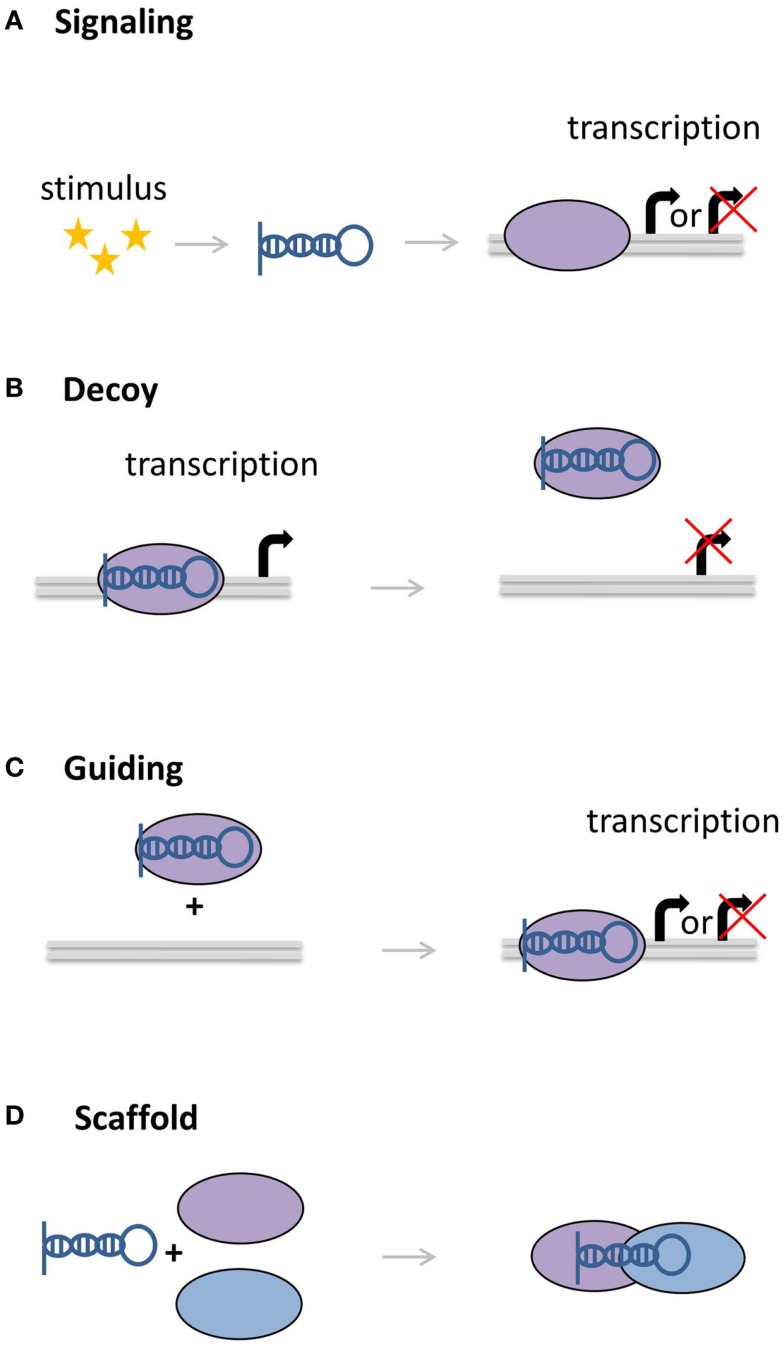
**Molecular functions of lncRNAs**. **(A)** LncRNAs can act as signaling molecules, affecting the expression of genes in response to a stimulus. **(B)** LncRNAs can divert transcription factors or other proteins away from the DNA. **(C)** Other lncRNAs can recruit proteins, bringing them closer to target genes. **(D)** As scaffolds, lncRNAs can bring together multiple proteins to form complexes.

The GENCODE (V7) compendium has annotated over 13,000 human lncRNAs, of which only a fraction, however, has a known function (26). LncRNAs can have diverse molecular functions relayed by the molecules they interact with: mRNA, protein, miRNAs, or DNA (Figure 3) (33). These interactions can affect processes like transcription, translation, splicing, translation, or RNA degradation (34, 35). Chang and Rinn classified lncRNAs into four subclasses by their different functions (34). For example, lncRNAs can function as molecular scaffolds to bring proteins together in a complex, but they can also act as a signal for a specific biological condition or state, for instance cellular stress or temperature. The signal can subsequently activate or repress the expression of other genes. Another function lncRNAs can exhibit is that of being a decoy, in which they bind to other RNAs or proteins and interfere with their function. Finally, lncRNAs can guide protein complexes to targets, where they can act as activators or repressors of other genes. In addition to these four main functions, some lncRNAs can inhibit the function of miRNAs, thereby alleviating the downregulating effect of the miRNA on the gene expression (36–38). Note that it is also possible for lncRNAs to exert multiple of these functions.

## The Role of miRNAs and lncRNAs in the Immune System

It has been proposed that miRNA emerged as a primitive immune response against viral infection. The fact that they show remarkable conservation in animals and plants suggests they hold important biological functions. MiRNAs play important roles in cell physiology, as clearly demonstrated by the fact that Dicer knockout in mouse embryos is incompatible with life. Deleting or overexpressing individual miRNAs in mice offers the opportunity to study their roles in the immune response (39). Lineage-specific knockout (KO) of miRNAs in specific immune cell types results in severe perturbation of immune cell numbers, their composition, and function. These all points to miRNAs being essential for immune cell development, differentiation, function, and homeostasis (39–43).

*Dicer1* deletion in granulocyte-macrophage progenitors (derived from a myeloid-specific *CCAAT/enhancer binding protein-*α *(Cebpa)-Cre*-driven *Dicer1-*deleter mouse strain) resulted in changes in gene expression profiles, increased self-renewal ability of precursors in the bone marrow (BM), monocyte depletion, and myeloid dysplasia, underlining the essential contribution of miRNAs to myeloid development (44). The role of Dicer and miRNAs has also been demonstrated in natural killer (NK) cells. By using mice with conditional deletion of *Dicer1* or *Dgcr8* in NK cells, a reduced cellularity in the spleen was observed with a concomitant reduced frequency of splenic NK cells, but without alterations in T- and B-cell frequencies. *Dicer* and *Dgcr8* deficiency was associated with an increase in NK apoptosis and an impairment in NK activation, suggesting that miRNAs are required for NK homeostasis and function (45).

As discussed above, miRNAs can be packaged into exosomes that are subsequently secreted from the cell. It has been suggested that circulating miRNAs could act in intercellular communication, also in the immune system. For example, it was suggested that T-cells communicate with antigen-presenting cells (APCs) by a unidirectional transfer of exosomal miRNA. Another example is the transfer of miR-335 (downregulating SOX4, a progenitor cell transcription factor), which was correlated with the transfer of CD63 upon formation of the immune synapse (24). Furthermore, exosomes released from mature BM-derived DCs contain more *miR-125-5p*, *miR-146a*, and *miR-148*, which are negative regulators of pro-inflammatory factors in myeloid cells and DCs. Exosomes released by both immature and by mature BM-derived DCs contain *miR-34a* and *miR-21* (known to regulate the differentiation of hematopoietic precursors into myeloid DCs), as well as *miR-221* and *miR-222* (that prevent differentiation into plasmacytoid DCs). Such exosomes can be taken up by recipient DCs, and the packaged miRNAs can then be released to target known binding sites, as shown by 3′-UTR-luciferase experiments (46). Exosome-derived miRNAs have also been implicated in the progression of Epstein–Barr virus (EBV) infection. Exosomes containing EBV-derived miRNAs are released from infected B-cells and taken up by DCs, where the miRNAs can then downregulate the expression of genes encoding immune-stimulation factors (47). Together, these limited but suggestive data point to a role for miRNA-based intercellular communication, mediated by exosomes, in the immune system, which has implications for health and disease.

Because lncRNAs are not produced via a lncRNA-specific biochemical pathway, it is not feasible to generate general or lineage-specific lncRNA-knockout mice. The lack of evolutionary conservation of lncRNAs across species further complicates the study of their individual function. Nevertheless, several mouse knockouts have been generated for single lncRNAs. A landmark study described the generation of 18 mouse strains, all with one lncRNA deleted (48). Although the lncRNA candidates were not selected for immune cell specificity, the study revealed key roles for several individual lincRNAs in the viability and developmental processes of the mice and it also highlighted the importance of using *in vivo* models to reveal the biological significance and functional diversity of lncRNAs (48).

In the next section, we give examples of how key miRNAs play regulatory roles in the development and activation of the immune system and we summarize the much smaller body of evidence implicating lncRNAs in these processes.

### The role of miRNAs in the development of innate immune cells

**Figure 4:**
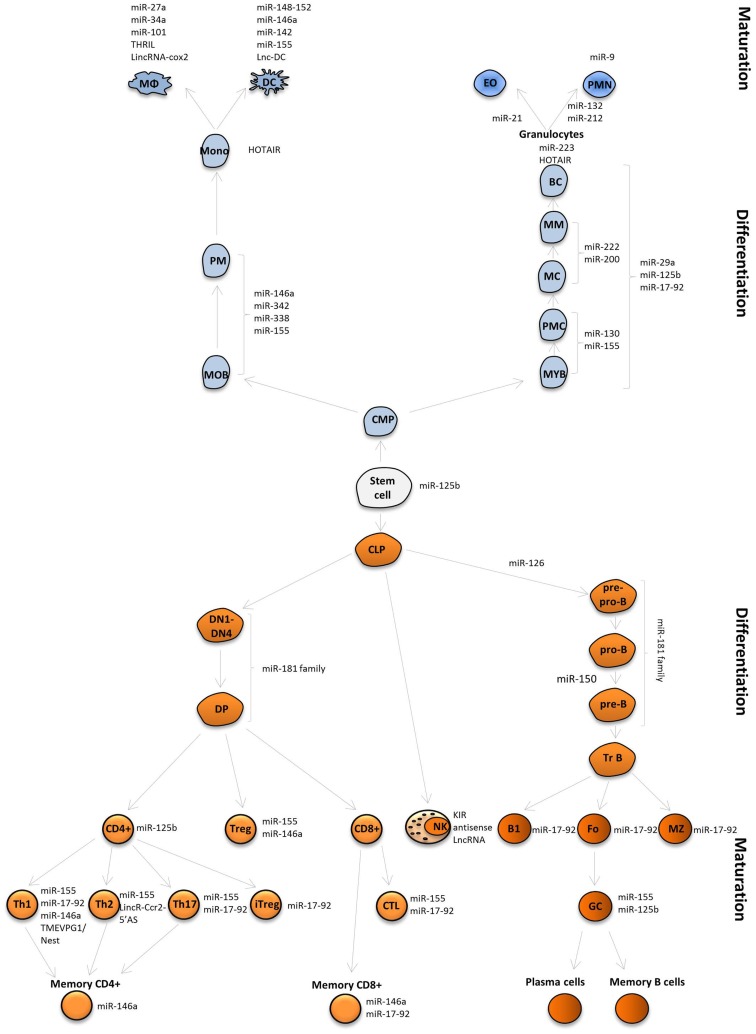
**MiRNAs and lncRNAs influence immune cell fate and function**. MiRNAs and lncRNAs were shown to modulate development and function the immune system. LncRNAs and selected miRNAs that are discussed in this review are depicted.

The innate immune system includes myeloid cells derived from hematopoietic stem cells (HSCs) and myeloid progenitors. These cells give rise to monocytes, which can develop into macrophages and DCs, and to granulocytes (neutrophils, eosinophils, basophils) through a series of developmental stages (myeloblast, promyelocyte, myelocyte, metamyelocyte, band cell or monoblast, and promonocyte) (Figure 4). One of the first studies of miRNA expression in normal human granulocytes reported sets of miRNAs that were subject to upregulation or downregulation at discrete maturation stages in neutrophil development. Although the majority of miRNA family members showed coordinated expression patterns, the expression of some miRNAs in the same cluster is not always synchronized. For example, the *miR-17-92/oncomir-1* cluster encompasses six miRNAs (*miR-17*, *-18a*, *-19a*, *-20a*, *-19b-1*, *-92a-1*). Among the cluster’s targets are antitumor, pro-apoptotic, and tumor suppressor proteins. HSCs and early progenitors in the BM express high levels of miRNAs from this cluster, whereas their expression is reduced during myeloid and lymphoid differentiation (49, 50). Of this cluster, *miR-20a* and *miR-92* are downregulated in metamyelocytes, *miR-18a, miR-19a* and *miR-19b* are downregulated in neutrophils, while *miR17-5p* gradually decreased from myeloblasts in the subsequent stages of development (51). In *miR-223* KO mice, it was shown that *miR-223* deletion leads to an increase in the number of granulocyte progenitors and neutrophil hyperactivity, suggesting that *miR-223* acts as a crucial regulator of granulocyte production and the inflammatory response (52).

*In vitro* overexpression or knockdown experiments of *miR-29a* or *miR-142-3p* in human leukemia cell lines showed that the miRNA overexpression promoted monocytic and myelocytic maturation, while blockage with antisense inhibitors promoted not only the expression of early progenitor markers but also reduced cell maturation, indicating these miRNAs play roles as regulators of normal myeloid differentiation. *MiR-29a* and *miR-142-3p* were both shown to target cyclin T2 (*CCNT2*), while they were also specifically targeting the cyclin-dependent kinase 6 (*CDK6*) gene (*miR-29a*) and the transforming growth factor β (*TGF*β) activated kinase 1/MAP3K7 binding protein 2 (*TAB2*) gene (*miR-142-3p*) (41).

An important group of miRNAs in myeloid biology is the *miR-125* family, consisting of *miR-125a*, *-125b1*, and *-125b2*. The family members target crucial factors involved in HSC survival and apoptosis (42). *MiR-125b* overexpression, instigated by transplanting fetal liver cells ectopically expressing *miR-125b* in mice, caused a lethal myeloproliferative disorder (43). In addition, enforced expression of *miR-125b* in BM chimeric mice promoted myelopoiesis. B-cell lymphoma-2 (Bcl-2) homologous antagonist/killer 1 (*Bak1*) and the signal transducer and activator of transcription 3 (*Stat3*) were proposed as possible target genes (53). Moreover, using a miRNA-sponge approach, it was shown that *miR-125b* can also regulate myelopoiesis in mice by targeting *Lin28A*, an important regulator of hematopoiesis (54).

In PU.1-deficient mice, the development of macrophages, granulocytes, and B-lymphocytes is impaired, revealing that the PU.1 transcription factor is involved in myeloid and lymphoid development (55). Several miRNAs, including the *miR-17-92* cluster, are activated by PU.1 to modulate macrophage development. In PUER cells (murine myeloid progenitors in which macrophage development can be supported on inducing a tamoxifen-inducible PU.1 transgene), it was demonstrated that macrophage differentiation requires downregulation of *miR-17-92* (49). Moreover, PU.1 may also regulate macrophage development by inducing *miR-146a*, *miR-342*, *miR-338*, and *miR-155* (56). *miR-142* is another miRNA involved in myeloid development. In *miR-142-*deficient mice, a reduction of CD4^+^ DCs is accompanied by a severe defect in their ability to prime CD4^+^ T-cells (57). MiRNA expression profiling during human monocyte differentiation has shown a decrease in levels of miRNAs, the *miR-17-92* cluster (*miR-17-5p* and *miR-20a*), as well as of *miR-106a* (a member of *miR-106a-363*, a paralog of the *miR-17-92* cluster), compared to early progenitors. One of the shared targets of *miR-17-5p*, *miR-20a*, and *miR-106a* is the runt-related transcription factor 1 (*RUNX-1*) gene, an important regulator of hematopoiesis (58).

Analysis of miRNA profiles in human BM precursors and neutrophils revealed that 135 miRNAs were differentially expressed between the myeloid developmental stages. For instance, high levels of *miR-130a*, *miR-155*, and *miR-146a* were observed in myeloblasts and promyelocytes, followed by a decrease in expression in more mature cells. Potential targets for these miRNAs include transcripts encoding members of the TGFβ signaling pathway, such as *TGF*β*-receptor 1 (TGF*β*R1)* and *TGF*β*R2, SMAD2*, *SMAD4*, and *SMAD5 (miR-130)*. Some miRNAs clustered with the intermediate stages of development (*miR-222*, *miR-200*, *miR 29a*), while others were associated with mature neutrophils (*miR-132*, *miR-212*). Among the predicted targets of the miRNAs listed above are transcripts encoding cell cycle regulators, such as *CDK2* (*miR-155*), or proteins associated with apoptosis such as apoptotic protease activating factor 1 (*APAF1)*, *CASP8*, and Fas-associated death domain (*FADD)*, which are targeted by *miR-132*, *miR-212* (59).

Another miRNA important in the development of the innate immune system is *miR-21*. It has been identified as one of the most highly upregulated miRNAs in allergic diseases and this is associated with high numbers of eosinophils, the main effector cells in allergic responses. In a report by Lu et al. (60), the role of *miR-21* was evaluated in a murine *ex vivo* culture system. By using RT-PCR, it was shown that during eosinophil differentiation *miR-21* was upregulated threefold from day 4 to day 14 in culture. Cultures derived from *miR-21^−/−^* eosinophil progenitor cells showed higher apoptosis than cultures from *miR-21*^+^*^/^*^+^ progenitor cells, suggesting that *miR-21* regulates the development of eosinophils by modulating eosinophil progenitor cell growth. In agreement with these findings, *miR-21^−/−^*mice showed reduced blood eosinophil levels, concomitant with a reduced capacity to produce eosinophils in the BM. Microarray analysis revealed the differential expression of genes involved in cell proliferation, cell cycle control, and the immune response (60).

### MiRNAs involved in innate immune cell activation

The best-known example of a miRNA involved in the activation of innate and adaptive immune cells is *miR-155*. It has also been implicated as a general and conserved feature of mouse and human DC activation by various Toll-like receptor (TLR) ligands (61, 62). Analyses of *miR-155*-deficient mice demonstrated that although the development of DCs was unaffected, *miR-155* is required for DC maturation and the ability to promote antigen-specific T-cell activation (61). DC maturation is also affected by *miR-150*, *miR-34a*, and *let-7i* by mechanisms that involve silencing c-Fos (*miR-155*), *Csf1r* (which controls M-CSF receptor expression; *miR-34a*), and suppressor of cytokine signaling-1 (SOCS1) (*let-7i*) expression (61–64). Another group of miRNAs, including *miR-146a*, *miR-148*, and *miR-142*, have been associated with downregulation of inflammatory pathways and modulation of DC maturation (65–67). *MiR-146a* controls DC cross-priming (by suppressing Notch1 expression and IL-12p70 production) and DC activation (by targeting TLR9, TLR2, interleukin-1 receptor-associated kinase 1 (IRAK1), and tumor necrosis factor (TNF) receptor-associated factor 6 (TRAF6) signaling) (65–68). Another important regulator of DC maturation is calcium/calmodulin-dependent protein kinase II (CaMKII), which is a target of three members of the *miR-148/152* family (*miR-148a*, *miR-148b*, and *miR-152*). These miRNAs downregulate CaMKII leading to reduced expression of MHCII, reduced cytokine production (IL-12, IL-6, TNF-α), and a reduced antigen-presenting capacity of DCs (66).

Micro-RNA profiling of macrophages stimulated with TLR ligands and cytokines has shown the involvement of several miRNAs in inflammation. After incubation of murine macrophages with lipopolysaccharide (LPS), poly I:C, or interferon β (IFN-β), the expression of *miR-9*, *miR-101*, *miR-155* was upregulated, while *miR-34* and *miR-27a* were downregulated to modulate the levels of important regulators of inflammation. The upregulated miRNAs target nuclear factor-κB1 (NFκB1, *miR-9*), mitogen-activated protein kinase 1 (MAPK1, *miR-101*), and JUN N-terminal kinase (JNK, *miR-155*), while the downregulated miRNAs target NOTCH1 (*miR-34a*) and STAT3 (*miR-27a*), and the production of pro-inflammatory cytokines such as TNF-α, interleukin-6 (IL-6), and IL-10 (69–73).

*MiR-9* was also associated with the response of polymorphonuclear cells (PMN) to TLR stimulation. It was interesting that out of the 365 miRNAs tested, *miR-9* was the only one upregulated in both human macrophages and PMN after LPS activation (69). In contrast, *miR-155*, *miR-146a*, *miR-146b*, *miR-187*, *miR-125a*, *miR-99b*, and *let-7e* appeared to be macrophage-specific, while *miR-196a* was PMN-specific. This underscores how some miRNAs are involved in the activation of multiple lineages of innate immune cells, while others play a more lineage-specific role (69).

Recent studies have shown that microorganisms can modulate miRNA expression and thus the immune response during infection, as a mechanism of immune evasion. *Mycobacterium tuberculosis* induces *miR-21* expression in macrophages and DCs. It was suggested that by targeting IL-12, *miR-21* modulates the Th1 immune response (74). *Leishmania* has also developed strategies to subvert the host macrophage response. On *Leishmania* infection of human macrophages *in vitro*, approximately 64 out of 365 analyzed miRNAs were found to be modulated. Enrichment analyses have revealed that several of these differentially expressed miRNAs are involved in the regulation of TLR and pro-apoptotic pathways (75). By using the murine model of *Toxoplasma* infection, an increase in the levels of the immune-miRNAs *miR-146a* and *miR-155* was observed in the brain of chronically infected mice compared with non-infected controls. Further assays in *miR-146* KO mice demonstrated that *miR-146* ablation promotes parasite control, resulting in long-term survival (76). MiRNA profile expression analyses of human macrophages infected with *Toxoplasma* showed that the *miR-17-92* cluster expression was significantly upregulated and that the levels of *miR-17-92* were closely related with a decrease in expression of the pro-apoptotic regulator Bim. Interestingly, the Bim 3’-UTR contains predicted binding sites for multiple miRNAs derived from the *miR-17-92* family. All the above evidence suggests miRNAs are involved in parasite persistence and modulation of apoptosis (77).

### Long non-coding RNAs in innate immunity

So far, most lncRNA studies have been performed in normal cellular development or in cancer (78–82), although the role of lncRNAs in hematopoiesis and the immune system is slowly starting to emerge. The lncRNA *HOTAIRM1*, located antisense to homeobox A1 (HOXA1) and HOXA2 of the HOXA gene cluster, is expressed specifically in the myeloid lineage (83). *HOTAIRM1* is upregulated during retinoic acid-driven granulocytic differentiation in NB4 promyelocytic leukemia cells, which are a model for granulocytic differentiation. Knockdown of *HOTAIRM1* prevents the expression of HOXA1, HOXA4, CD11b, and CD18, but not of the more distal HOXA genes and decreased myeloid differentiation.

The *KIR antisense lncRNA* was found to be expressed only in human embryonic stem cells and other cell types with stem cell properties (84). KIR genes encode class-I MHC receptors expressed on human NK cells. *KIR antisense lncRNA* overlaps with exons 1 and 2 of the protein-coding KIR gene, as well as with an upstream proximal promoter region of the KIR genes. Overexpression of the lncRNA in NK cells was found to decrease the expression of the KIR protein-coding gene. Wright et al. speculated that the KIR genes are silenced in NK progenitors so that they are not able to influence the process of NK cell differentiation (84). As the KIR distal antisense promoter contains myeloid zinc finger-1 (MZF-1)-binding sites, it is assumed that this transcription factor regulates the expression of the *KIR antisense lncRNA*. MZF-1 is a transcriptional regulator that is able to activate transcription in cells of hematopoietic origins, whereas it can repress transcription in other cell types. However, the precise mechanism of the regulation of *KIR antisense lncRNA* expression is unknown.

*Lnc-DC*, exclusively expressed in conventional human DCs (85), was found to induce the nuclear translocation of STAT3. The proposed mechanism of action for this lncRNA is to prevent SHP1 from binding to phosphorylated STAT3 and dephosphorylating it, thereby preventing its dimerization and translocation to the nucleus. This is an example of a lncRNA affecting cellular differentiation by a mechanism that takes place in the cytoplasm.

In another study, 54 mouse pseudogene lncRNAs were found to be induced by TNF-α (86). One of these, *Lethe*, functions as a negative feedback signal that inhibits NF-κB. Its expression is increased when TNF-α activates NF-κB, after which *Lethe* binds to NF-κB and prevents it from binding to DNA, thereby inhibiting the expression of inflammatory proteins, such as IL-6, IL-8, and superoxide dismutase 2 (SOD2).

A whole-transcriptome profiling of mouse macrophages stimulated with different TLR ligands uncovered dozens of expressed lncRNAs (87). Activation by the synthetic bacterial lipopeptide Pam3CSK4, a TLR2 ligand, resulted in the expression of 62 lncRNAs. One of them, *lincRNA-Cox2*, acts as a key regulator of the inflammatory response by mediating both activation and repression of several immune genes. In response to TLR2-stimulation, *lincRNA-Cox2* induces the expression of hundreds of genes, including Tlr1, Il-6, and Il-23a. *LincRNA-Cox2*-mediated repression of target gene expression was found to require the interaction of *lincRNA-Cox2* with hnRNPA/B and hnRNPA2/B1, repressing the transcription of immune cells.

Stimulation of human THP1 macrophage cells by a synthetic lipopeptide ligand of TLR2 induced 159 lincRNAs (88). One of these, TNFα and hnRNPL-related immunoregulatory lincRNA (*THRIL*), form a complex with hnRNPL. This complex can bind the promoter of TNFα and regulate its transcription. Microarray analysis showed that *THRIL* is required for the expression of various immune genes, including cytokines and other regulators of TNFα expression, including IL-8, C-X-C motif chemokine 10 (CXCL10), chemokine (C-C motif) ligand 1 (CCL1), and the colony stimulating factor 1 (CSF1). *THRIL* expression was also reported to be correlated with the severity of symptoms in patients with Kawasaki disease, an autoimmune disease mostly seen in children.

*NEAT1 (nuclear enriched abundant transcript 1)* is a lncRNA that was shown to be essential for the formation of paraspeckles. Paraspeckles are nuclear bodies found in mammalian cell nuclei and it has been proposed that they play a role in several biological processes, including cellular differentiation and the stress response (89). It has been shown that *NEAT1* is induced by viral infection as well as by poly I:C stimulation and that, in response to such a stimulus, *NEAT1* binds to paraspeckle protein splicing factor proline/glutamine-rich (SFPQ). This complex binds to and regulates the expression of several antiviral genes, including *IL-8*, which induces the formation of paraspeckles (90).

## The Role of miRNA in the Development of Cells of the Adaptive Immune System

### miRNAs in T-cell development and activation

Micro-RNAs have been shown to be crucial for both immune system development and its functioning. MiRNAs that are characteristically enriched in HSCs and progenitor cells are *miR-125a-5p*, *miR-125b-5p*, *miR-155*, *miR-130a*, *miR-196b*, *miR-99a*, *miR-126-3p*, *miR-181c*, *miR-193b*, *miR-542-5p*, and *let-7e* (91). Their expression changes during immune cell development.

Some miRNAs are selectively expressed in specific stages of immune cell development, whereas others are more broadly expressed. Profiling studies showed that there are miRNAs, which are preferentially upregulated in lymphocytes. The *miR-181* family is abundant expressed in developing BM B-cells and thymocytes.

The importance of miRNAs in T-cell biology has been extensively studied in mice with conditional *Dicer1* deletion. Conditional deletion of *Dicer1* in T-cell precursors using *Lck-Cre* demonstrated that Dicer is necessary for the generation and survival of normal numbers of αβ T-cells (92). *Cd4-Cre*-mediated deletion did not affect the viability of Cd4+ T-cells, but the numbers of Th1 and Th2 cells were significantly reduced, resulting from both decreased proliferation as well as from increased apoptosis. Dicer-deficient Cd4+ cells have been described as more prone to differentiate into Th1 cells and Dicer-deficient cells cannot repress Inf-γ upon Th2 stimulation (93).

Profiling of different stages of T-cell development, starting from the double negative 1 (DN1) thymocyte stage, reveals that miRNA profiles are similar for cells with similar developmental status (94). DN3 and DN4 populations cluster together based on their miRNA expression profiles, as do mature single positive Cd4+ and Cd8+ cells. DN1 cells are more similar to DN3 and DN4 cells. Nevertheless, expression of individual miRNAs changes depending on their developmental stage. Each of the stages is characterized by elevated expression of at least one miRNA or miRNA family. In DN1 cells, *miR-21*, *miR-29b*, *miR-342*, *miR-221*, and *miR-223* are elevated and *miR-16, miR-181a*, and *miR-15b* are decreased. *MiR-191* is upregulated and *miR-142-3p* is downregulated in DN3 cells. *MiR-142-5p*, *miR-20a*, *miR-16*, and *miR-128b* are increased, whereas *miR-150* is decreased in DN4 cells. In double positive (DP) cells, expression of *miR-92*, *miR-181a*, *miR-181b*, and *miR-350* are enhanced, while in Cd4+ cells, *miR-669c* and *miR-297* are elevated. In Cd4+ and Cd8+ cells, *miR-128* is abundant.

It is interesting that, on activation of Cd4+ T-cells, Ago2 ubiquitination and consequentially its proteosomal degradation is induced, leading to global miRNA downregulation. Moreover, naive T-cells display reduced levels of Ago2 and differentiate more rapidly. These findings led to the hypothesis that the decrease in the miRNA pool on T-cell activation allows the expression of genes regulating CD4+ T-cell differentiation and facilitates the gain of T-cell effector functions (95). Although these are global effects on the miRNA pool, some miRNAs can be picked out that play key roles in T-cell biology. For example, the *miR-181* family is upregulated in DP cells and its family member *miR-181a* decreases the expression of Cd69, T-cell receptor α (Tcrα), and Bcl-2 (94).

Another key player is *miR-125b*. This miRNA is part of the *miR-99a/100*~*125b* tricistrons, located on human chromosomes 11, 19, and 21. The tricistron on chromosome 21, encompassing *miR-99a/let-7c/miR-125b-2*, is highly expressed in HSCs and is responsible for maintaining stem cell properties (96). In human naive CD4+ T-cells, *miR-125b* downregulates proteins that are critically involved in T-cell differentiation: *IFN-*γ, *IL-2RB*, *IL-10RA*, and *PR domain zinc finger protein 1* (*PRDM1*, encoding B lymphocyte-induced maturation protein-1, BLIMP-1). Overexpression of *miR-125b* inhibits the differentiation of naive T-cells into effector cells (97). This miRNA is an example of one that affects various stages of immune cell differentiation in different immune cell lineages.

T-cell activation leads to highly elevated expression of *miR-155* (98). Experiments conducted on Cd4+ T-cells isolated from mice deficient for *Bic*, the primary transcript encoding *miR-155*, uncovered elevated Th2 polarization, and Th2 cytokine production (Il-4, Il-5, and Il-10) in these cells. This effect is mediated by upregulation of c-Maf, a Th2-specific transcription factor known to induce the expression of Il-4/5/10 (99). Th1 and Th17 responses are also regulated by *miR-155*. Transfection with *miR-155* promotes, whereas *miR-155* inhibition decreases, the number of Th1 and Th17 cells in mice with experimental autoimmune encephalomyelitis (EAE) (100). Mice lacking *Bic* also display decreased levels of regulatory T-cells (Tregs) in the thymus and in the periphery while the function of these cells *in vitro* is not affected. This indicates that *miR-155* is required for Treg development (101). Characteristically, *miR-155* expression is induced in Tregs by forkhead box P3 (Foxp3), while one of the main targets of *miR-155* in Tregs is Socs1. When *miR-155* is high, Socs1 is low, which contributes to maintaining the competitive fitness and proliferative potential of Tregs (102).

The *miR-17-92* cluster is a master switch involved in the differentiation into Th1 and Th17 cells. Experiments conducted in *cd4-cre*-driven *miR-17-92* conditional KO mice demonstrated that Th1 development is critically controlled by *miR-17* and *miR-19b*, which target *Tgf*β*rII* and *cyclic AMP-responsive element binding protein 1 (Creb1, miR-17*), and *Pten* (*miR-19b*). Together, these two miRNAs enhance T-cell proliferation and Inf-γ production, protect from activation-induced cell death, and repress induced Treg (iTreg) differentiation. Interestingly, *miR-18a* of the same cluster antagonizes the pro-Th1 effect of *miR-17* and *miR-19b* through elevation of activation-induced cell death and inhibition of proliferation (103). Subsequent experiments conducted on T-cells isolated from conditional *miR-17-92*-depleted mice (that had been retrovirally transduced with selected miRNAs from the *miR-17-92* cluster), showed that *miR-17* and *miR-19b* were also the miRNAs promoting Th17 differentiation. This is mediated by *miR-17*-induced downregulation of Ikaros family zinc finger 4 (Ikzf4) and the downregulation of Pten by *miR-19b* (104).

Another miRNA regulating the adaptive response is *miR-146a*. Profiling studies in mice showed that *miR-146a* expression is high in Th1 cells and low in naive T-cells and Th2 cells (105), but very high in Tregs (106). Level of *miR-146a* is also elevated in human memory cells (both in CD4 and CD8 memory cells). *MiR-146* expression is induced on TCR stimulation and is regulated by NF-κB and the c-E26 transformation specific (c-ETS) transcription factor. It was suggested that *miR-146a* exerts its regulatory function by targeting FADD, leading to a decrease in apoptosis. On TCR stimulation, activator protein-1 (AP-1) activity and IL-2 production are induced, but *miR-146* targets both of them, thereby enabling *miR-146a* to affect the duration of T-cell activation phases (107).

As can be expected, the importance of miRNAs in CD8+ biology has been well studied. *Cd4-Cre*-induced Dicer deletion in mice leads to reduced development of peripheral Cd8+ cells due to decreased cell survival and defective migration out of the primary lymphoid compartment (93). As was also the case for CD4+ cells, a decrease in Dicer (and therefore in the miRNA pool) leads to differentiation, characterized by increased levels of perforin, granzymes, and effector cytokines that are usually targeted by *miR-139* (targets Eomes and perforin) and *miR-150* (targets Cd25) (108). Another study reported that *miR-15b*, *miR-150*, *miR-24*, and *miR-27a* were increased in Cd8+ cells (94).

*MiR-155* expression also plays an important regulatory role in CD8+ cells. It is most highly expressed in primary effector Cd8+, shows intermediate expression in effector memory Cd8+ T-cells, and low expression in naive Cd8+ and central memory Cd8+ cells. Its role in the antiviral response of Cd8+ cells was demonstrated in *miR-155*-deficient mice, which are characterized by an attenuated antiviral response due to a diminished response of Cd8+ cells. On the contrary, overexpression of *miR-155* in mice enhanced the antiviral Cd8+ response (109).

In short-lived effector CD8+ cells, *miR-17-92* is upregulated in contrast to memory cells. Experiments on mice with a conditional gain or loss of *miR-17-92* expression in mature Cd8+ cells after activation (controlled by the human Granzyme B promoter) revealed that *miR-17-92* regulates Cd8+ expansion and the balance between effector and memory differentiation. *MiR-17-92* overexpression elevates the differentiation into terminal effector cells and concomitantly decreases the formation of polyfunctional lymphoid memory cells. As *miR-17-92* overexpression correlates with downregulation of Pten, and in consequence induces the PI3K (phosphoinositide 3-kinase)-Akt-mTor (mammalian target of rapamycin) pathway, here too it was suggested that this could be the main pathway involved in regulating cellular proliferation. In contrast, conditional deletion of *miR-17-92* leads to attenuated proliferation of antigen-specific cells, increased Il-7Rα and Bck-2 expression, and faster acquisition of memory cell properties (110).

### miRNAs in B-cell biology

Experiments with conditional Dicer-KO mice in early B-cell progenitors (*Mb1-Cre* drives deletion starting from the pro-B-cell stage) resulted in a block in B-cell development at the transition from pro- to pre-B-cell stage (111). Conditional deletion of *Dicer-1* in later stages of B-cell development showed that miRNAs are also critically important for the transition from transitional B-cells to germinal center (GC) or follicular (Fo) B-cells (112). Conditional deletion of Dicer in activated B-cells [*activation-induced cytidine deaminase (Aicda-cre)*] confirmed that Dicer is essential for GC B-cell generation. The ablation of Bim partially rescued this effect (113).

The analysis of different cell subtypes during B-cell development in mice underscored the highly regulated, developmental stage specific, expression of miRNAs. Hierarchical clustering of BM and spleen B populations leads to a perfect recapitulation of the B-cell developmental pathway. This study found the population with the most distinct miRNA profile was that of fraction A (FrA) B-cells, characterized by expression of *miR-2138*, *-542-3p*, *-500*, *-1959*, *-221*, *-1965*, *-1900*, *-1893*, *-501-5p*, and let-7f*. FrA cells have been reported to still retain the capacity to differentiate into T-cells, while Pax5 and Cd19 expression is induced in the next developmental stage leading to FrB/C B-cells. Among the miRNAs not expressed in the BM are *miR-150* and *miR-155*. These miRNAs start to be expressed in transitional B-cells and are most highly expressed in mature B-cells in the spleen (50).

In this B-cell lineage, specific or more broadly expressed miRNAs also control the differentiation and activation. *MiR-126* expression decreases during B-cell maturation. Injecting *miR-126*-overexpressing HSC/progenitors cells into lethally irradiated mice showed that *miR-126* induces the differentiation of B-cell myeloid progenitors. One of the genes regulated by *miR-126* in this process is insulin regulatory subunit-1 (Irs-1) (114).

*MiR-181* is preferentially expressed in the B-cells in the BM. Its overexpression in HSCs and progenitor cells leads to increased levels of B-cells. Moreover, ectopic expression of *miR-181* in Lin^-^ BM cells transplanted into sublethally irradiated mice leads to an increase in B-cells, with a concomitant decrease of T-lymphoid cells (115).

Mice without *miR-17-92* (a *Cre-deletor* mouse strain with *Cre* controlled by the human β*-actin* promoter) die shortly after birth. Analysis of fetal liver cells from these mice showed that the frequency of HSCs and the number of early progenitors was not affected, but that the number of pre-B-cells was significantly reduced. In *miR-17-92*-deficient adult mice, marginal zone (MZ), Fo, and newly formed B-cells in spleen (as well as peritoneal B1a and B1b cells) are reduced, whereas the relative number of transitional B-cells was not altered. Moreover, in these mice, the frequency of red blood cells, granulocytes, and monocytes was also not altered (116). In contrast, another study showed that B-cell-specific *miR-17-92* overexpressing mice develop B-cell lymphomas due to the downregulation of negative regulators of the PI3K pathway [Pten and PH domain and leucine-rich repeat protein phosphatase 2 (*Phlpp2*)] and the NF-κB pathway (*Cyld, A20, Itch, Rnfl1, and Tax1bp1*), as well as due to the downregulation pro-apoptotic protein Bim and cell cycle regulator E2F3. Together, this results in constitutive activation of pro-survival pathways (117).

*MiR-150* is expressed in mature B-cells but not in BM B-cells. Premature overexpression of *miR-150* in HSCs showed that *miR-150* blocks the generation of mature B-cells by preventing the transition of pro-B-cells to pre-B-cells, but not the development of T-cells, granulocytes, or macrophages. The main target involved in this mechanism is c-Myb (118, 119).

*MiR-155* controls the GC response at least partially via regulation of cytokine production. Mature *miR-155^−/−^* cells isolated from spleens are deficient in Tnf and lymphotoxin-α (Lt-α) production (120). *MiR-155* was also shown to be critically involved in isotype switching, as reduced extrafollicular and GC responses, and a concomitant lack of high-affinity IgG1, were observed in the absence of *miR-155* (121). *MiR-155* is often overexpressed in B-cell lymphomas including DLBCL (122), and *E*μ*-enhancer* driven *miR-155* overexpression leads to lympho-proliferative disease followed by B-cell malignancy (123).

Finally, *miR-125b* appears to inhibit GC B-cell differentiation by targeting BLIMP-1 and IFN regulatory factor 4 (IRF4); it is essential for the post-GC plasma B-cell differentiation (124).

## The Role of Long Non-Coding RNA in the Adaptive Immune System

Mouse CD8 + T-cells were found to specifically express hundreds of lncRNA genes. Many of these are specific for lymphoid cells and their expression was dynamically changed during lymphocyte differentiation or activation (125). A subset of 39 lncRNAs appear to be precursor transcripts to small regulatory RNAs (miRNAs and small interference RNAs, siRNAs), suggesting that some lncRNAs function via smaller RNA species.

The dynamic nature and cell-specific lncRNA expression during mouse T-cell differentiation was demonstrated by RNA-seq analysis of 42 T-cell subsets (from early T-cell progenitors to terminally differentiated T helper subsets, at multiple time points during differentiation) (126). This led to the identification of 1,524 lincRNAs, most of which are located adjacent to key proteins that regulate the immune system. Knockdown of one of these lncRNAs, *LincR-Ccr2-5’AS*, led to deregulation of its neighboring chemokine receptor genes and prevented Th2 migration into the lung tissue (126).

In 2003, the *Tmevpg1* gene was shown to control the persistence of Theiler’s virus in the mouse central nervous system (127). Both the mouse gene and its human ortholog, *TMEVPG1/NeST*, encode a non-coding RNA located in a cluster of cytokine genes, including the IFN-γ gene, and it was suggested to be involved in controlling IFN-γ expression (128). Both the mouse and human lncRNAs are expressed in Th1 cells and depend on Stat4 and T-box expressed in T-cells (T-bet), two transcription factors regulating Th1 differentiation (128). Comparison of mouse strains with and without the capacity to clear Theiler’s virus revealed that mice that cannot clear the infection express *Tmevpg1* to a higher level, concomitantly with increased IFN-γ synthesis and enhanced resistance to *Salmonella enterica* infection (129). These results indicate that lncRNA *TMEVPG1/NeST* regulates IFN-γ expression and plays an important role in the susceptibility to viral and bacterial infections.

## The Role of miRNAs and lncRNAs in Autoimmune Diseases

### Celiac disease

Celiac disease (CeD) is characterized by a severe inflammatory reaction to gluten peptides derived from grain storage proteins; it occurs in patients with a susceptibility genotype. Besides sharing a number of phenotypic characteristics with inflammatory bowel disease (IBD), CeD also shares multiple genetic susceptibility loci with IBD (130). So far, there are limited data on the involvement of miRNAs in CeD and there are no publications on the role of lncRNAs in this autoimmune disease. The lack of a suitable animal model for CeD makes it impossible to study the role of specific miRNAs *in vivo*. However, profiling of miRNA expression in small intestinal biopsies from patients with active CeD versus controls showed that *miR-449*, *-492*, *-644*, *-503*, *-196a*, *-504*, *-500*, and *-330* were differentially expressed in CeD patients, with *miR-449* as the most upregulated miRNA. Putative targets of *miR-449* include mRNAs encoding proteins involved in the NOTCH signaling pathway. In agreement with this, the expression of the inflammation regulator, NOTCH1, was found to be decreased in the small intestine of CeD patients, suggesting that miRNAs can also control inflammation in CeD (131). Indirect evidence for the involvement of particular miRNAs in CeD was found by analyzing genome-wide association study (GWAS) data. Kumar et al. have described how CeD-associated SNPs may actually affect the 3′-UTR of *IRF4*, *PTPRK*, and *ICOSLG* and suggested that these might change miRNA-binding sites (132).

### Inflammatory bowel disease

Inflammatory bowel disease includes Crohn’s disease (CD) and ulcerative colitis (UC) (133). Recent GWAS and meta-analyses have identified 163 common risk loci for IBD and 47 unique risk loci associated with UC (134). Although the cause of IBD is unknown, there is evidence to suggest that an abnormal immune response to intestinal flora leads to this disease in genetically susceptible individuals. There have been various miRNA profiling studies published on IBD and the miRNA profiles in tissues or serum of UC and CD patients at different stages underscore the importance of miRNA as key regulators of the immune response in this disease (130, 131). Circulating miRNAs in serum were suggested as useful biomarkers for CD diagnosis. MiRNA RT-PCR revealed a set of 11 miRNAs that were significantly elevated in CD patients, but not in the serum of controls or in the serum of patients with active CeD (135).

The first report of miRNA expression in colonic mucosa samples from IBD patients identified 11 miRNA differentially expressed in active UC patients versus controls (136). Since then, the number of miRNAs linked with IBD has increased gradually (137). Several reports have demonstrated the alterations in expression of miRNAs involved in modulating different aspects of the innate and adaptive immunity, such as *miR-21*, *miR-29a*, *miR-150*, and *miR-155* (136, 138, 139). Recently, by using microarray-based miRNA profiling of colonic mucosal biopsies, five miRNAs were shown to be upregulated in patients with active UC compared to quiescent UC, CD patients, and controls. In addition, expression of two miRNAs, *miR-125b-1* and let-7e*, was enhanced in patients with quiescent UC compared with active UC, CD patients, and controls, supporting the utility of miRNAs as biomarkers to distinguish the different IBD stages (140). An interesting point is that SNP rs2910164, which has been associated with susceptibility to CD, has also been linked to *miR-146a* (141). Subsequent reports have identified other miRNAs that may affect the control of inflammation during IBD. *MiRNA-155*, which has been associated with T-cell, B-cell, and innate cell function, was detected in the blood of CD and UC patients, but not in that of healthy controls (142). RT-PCR has revealed an upregulation in *miR-21* levels in mucosal tissue and serum and UC patients (143). Further, *in vitro* analyses demonstrated that overexpression of *miR-21* in mucosa from UC patients and in the Caco-2 cell model resulted in impaired tight junction formation and decreased barrier function, suggesting a pathogenic role for *miR-21* in UC (143). Using the murine model of dextran sulfate sodium (DSS)-induced colitis, it was found that overexpression of *miR-146b* (by expression vector) or ablation of *miR-21* (*miR-21* KO mice) reduces intestinal inflammation and restores epithelial barrier function by activating NF-κB (*miR-146b*) or by negatively regulating RhoB (*miR- 21*) (144, 145).

The involvement of other inflammatory pathways and processes regulated by miRNAs was also proven to be important in IBD pathogenesis. Several studies have shown that the pattern recognition receptor NOD 2 is upregulated by *miR-146* or downregulated by *miR-122*, while the colonic leukocytic trafficking is regulated by *miR-141* (146–148).

More studies have led to an understanding of the role of miRNAs on carcinogenesis, since IBD has been well established to be a predisposing condition for colorectal carcinoma (CRC). For instance, the levels of *miR-143* and *miR-145* were downregulated in UC patients compared with normal controls. Among the putative targets of these miRNAs are proteins associated with cell cycle regulation, such as K-RAS, API5, MAPK kinase-2 (MEK-2), and IRS-1 (149). A recent study identified *miR-224* as one of the most upregulated miRNAs during the transition from IBD to IBD-associated CRC. *In silico* analysis and functional assays confirmed that *miR-224* targets the cell cycle regulator p21, which could suggest the involvement of *miR-224* in IBD-associated carcinogenesis (150). Other studies have demonstrated a dysregulation of *miR-21* and *miR-155* during active IBD in IBD-dysplastic lesions (151, 152).

In conclusion, these inflammation-related miRNAs target important regulators of carcinogenesis, such as programed cell death 4 and mismatch repair elements, which could provide a biochemical link from IBD to cancer development (151, 152).

Because of their specific expression profiles, miRNAs are considered useful biomarkers for IBD diagnosis and as predictors of disease progression (153). *MiR-122*, *miR-17*, and *let-7e* were found to be altered during the progression of IBD (147), while a set of studies on immune-mediated diseases (including IBD) highlighted miRNAs as promising indicators of response to immunosuppressor treatment (20).

However, the role of lncRNA in the pathogenesis of CD remains elusive. A GWAS study identified *leucine-rich repeat kinase-2 (LLRK2)*, which is part of a complex including the large non-coding RNA repressor of NFAT, as associated with CD. In line with this, when wild-type mice were sublethally irradiated and reconstituted with *Lrrk2*-deficient hematopoietic cells, they were more susceptible to DSS-induced colitis. This suggests that *LLRK2* deficiency increases UC severity (154). In addition, high levels of lncRNA *DQ786243* were found in the blood of patients with CD. Subsequent overexpression of *DQ786243* in Jurkat cells showed a correlation between the lncRNA and the expression of the Foxp3 regulator, CREB, suggesting that *DQ786243* is involved in inflammation control and CD pathogenesis (155).

### Multiple sclerosis

Multiple sclerosis (MS) is an autoimmune, demyelinating neurodegenerative disorder, mostly affecting adults. Most often, the disease first manifests itself as relapsing-remitting MS (RR-MS), which typically progresses into secondary progressive MS (SP-MS) after 10–25 years. It is thought that RR-MS is more pro-inflammatory in nature, while SP-MS is mostly associated with neurodegeneration, although there is a small percentage of patients who present with neurodegenerative symptoms, without any evidence for former pro-inflammatory episodes. They have an aggressive form of MS called primary-progressive MS (PP-MS) (156). Multiple studies suggest the involvement of miRNAs in MS and changes in miRNAs have been detected in circulation, in brain tissue, in immune cells, and in CSF. Several miRNAs associated with the immune response have also been linked with MS, including miRNAs regulating the Th1 response, such as *miR-155* (100) and *miR-29* (157), but also Th17-associated miRNAs [*miR-155* (100), *miR-17-92* (104), *miR-132* (158), and *miR-326* (159)]. Of interest is that the level of *miR-326* in peripheral blood lymphocytes was suggested to distinguish between the relapsing and remitting phases of MS (160).

One of the miRNAs important in MS pathology is *miR-155*, the expression of which is elevated in RR-MS patients during relapse, as well as in the murine EAE model. In EAE mice, delivery of *miR-155* stimulates the development of inflammatory Th17 and Th1 cells, whereas applying miR-155 inhibitors reduces these processes (100).

The immune response in MS in the brain may be also regulated indirectly by changes in the blood–brain barrier (BBB) permeability. Elevated levels of *miR-155* in the neurovascular part of the brain of MS patients as well as in EAE mice are linked with a decreased function of the BBB. The potential targets of *miR-155* suggested to be involved in BBB function are focal adhesion proteins (DOCK-1, dedicator of cytokinesis-1, and Syntenin-1) and proteins involved in cell–cell interaction (Annexin-2 and Claudin-1) (161).

Another miRNA that plays a crucial role in regulating the function of the BBB, and consequently in regulating the immune cell influx in the brain, is *miR-125a-5p*. Overexpression of this miRNA elevates barrier tightness, with thicker and more continuous tight junctions formed by vascular endothelial-cadherin (VE-cadherin) and zona-occludens-1 (162).

MiR-92a-1* is reported to be increased specifically in the plasma of RR-MS patients compared to healthy controls or SP-MS patients. Furthermore, its level correlates negatively with disease duration and disability score. Elevated levels of *miR-92* can also distinguish RR-MS from another neuronal disease, amyotrophic lateral sclerosis. Using ingenuity pathway analysis (IPA), it was predicted that *miR-92* affects CD40 signaling by targeting CD40 directly (163).

*Let-7a* expression is diminished in SP-MS patients compared to healthy controls. IPA analysis led to the suggestion that *let-7a* targets TLR4 and TLR9, as well as IL-12RB2 and TGF-βR1 (163). *Let-7a* also regulates neuronal differentiation (164).

*MiR-145* appears to decrease during the transition from RR-MS to SP-MS and is negatively correlated with disability score (163). This miRNA is known to regulate insulin-like growth factor 1 (IGF-1) signaling (IGF-1R, IRS1, and IRS2) (165).

Long non-coding RNAs have been shown to be involved in the development of neurodegenerative disorders, like β*-secretase-1 (BACE1)-AS*, the *brain cytoplasmic RNA* (*BC200 RNA*) in Alzheimer’s disease, and the *brain-derived neurothrophic factor* (*BDNF)-AS* in Huntington’s disease (166). *BACE1-AS* elevates the protein level of BACE1, an enzyme involved in generating β-amyloid, by binding and stabilizing BACE1 mRNA (167). The level of *BC200 RNA* is elevated in the brains of Alzheimer’s patients (168) and this lncRNA was shown to interact with proteins participating in mRNA transport in neurons (166). *BDNF-AS* inhibits the expression of BDNF, a growth factor promoting neuron maturation and survival (169, 170). It is not yet known whether these lncRNAs are also involved in MS.

Impaired remyelination and neuron apoptosis are two hallmarks of the neurodegenerative phase of SP-MS. Interestingly, the level of *the small non-coding RNA 715 (sncRNA715)*, which inhibits oligodendrogial myelin basic protein (MBP) translation, is elevated in demyelinated chronic lesions in MS patients (171). In contrast, remyelination can be affected by exosomes released by DCs. Stimulating DCs with low levels of INF-γ causes the release of exosomes enriched in miRNA involved in the regulation of myelin production (*miR-219*) and anti-inflammatory response (*miR-181a*, *-451*, *-532-5p*, and *-665*) compared to exosomes released from unstimulated DCs. This, in turn, increases myelination and reduces oxidative stress in hippocampal slice cultures. Moreover, intranasal delivery of these exosomes elevates the central nervous system myelination *in vivo* (172). LncRNAs have also been implicated in the regulation of apoptosis, and because neuron apoptosis is important in MS, the role of lncRNA in apoptosis in MS should be investigated further (173).

Two MS SNPs are associated with miRNA genes (*miR-223*; rs1044165 and *miR-23*; rs3745453) and the expression of these miRNAs was investigated in MS patients (174). *MiR-223* is important for the innate immune system, e.g., affecting the non-canonical NF-κB pathway during macrophage differentiation (175), whereas *miR-23a* promotes myelination by elevating oligodendrocyte differentiation and myelin synthesis (176). Both *miR-223* and *miR-23a* are decreased in serum from RR-MS patients. However, the expression of *miR-233* is increased in peripheral blood mononuclear cells (PBMCs) from both RR-MS as well as PP-MS patients, whereas *miRNA-23a* is upregulated only in PBMCs from RR-MS patients (174). Thus far, no eQTL-effect of both SNPs on the respective miRNAs has been found.

Another study predicted that SNP rs28366, which is in moderate linkage disequilibrium (*r*^2^ = 0.4) with MS risk variant rs17066096, might affect the binding of *miR-2278* and *miR-411-5p*- to the 3′-UTR of *IL22RA2*. However, although these miRNAs did indeed bind to the 3′-UTR in an *in vitro* assay, the SNP did not affect the binding (177).

## Perspectives

Integration of autoimmune disease-associated SNPs with data on the functional regions in the genome shows that ~90% of these SNPs intersect with regulatory regions in the DNA (enhancers, promoters) rather than with protein-coding regions (exons) (29, 130). Autoimmune disease-associated loci often encompass genes known to play critical roles in immunological diseases. In these common illnesses, characteristically dozens of disease-associated SNPs have been identified, each predisposing moderately to disease. The general consensus for these immune-related diseases is that many SNPs with modest effects converge on a limited number of biological pathways and it is the sum of these effects that influences the phenotype to a large extent.

In the last two decades, thousands of regulatory RNA genes have been identified that also regulate gene expression. In fact, the total number of known miRNA genes plus lncRNA genes is now more than 15,000 and this is starting to approach the number of known protein-coding genes (~22,000). There is convincing evidence that autoimmune SNPs also affect miRNA and lncRNA biology. SNPs have been identified that locate to miRNA-binding sites and some SNPs appear to regulate the expression of lncRNAs (32, 132, 174, 177). This means that to fully understand the changes to transcriptional programs that contribute to disease, we need to take into account multiple gene-regulatory pathways, involving classical transcription factors, miRNAs, and lncRNAs. This situation is further complicated by the fact that these pathways, and the molecules involved in them, not only regulate other molecules within their own class, but also molecules of other classes. In the case of miRNAs and lncRNAs, for instance, it has already been reported that lncRNAs can be targeted by miRNAs, that lncRNAs can encode miRNAs, that lncRNAs and miRNAs can compete for targets, and that some lncRNAs can function as miRNA sponges (178).

With advances in next generation sequencing technology and the anticipated reduction in the cost of using these techniques, many more regulatory RNAs (and perhaps even novel classes of regulatory RNAs) are likely to be discovered. If we focus on discovering novel miRNAs and lncRNAs, we should take into account that the expression of both classes of regulatory RNAs is more cell-type- and stimulus-dependent than the expression of protein-coding genes. This has two consequences: (1) for regulatory RNAs affecting other RNA species, both the regulator and the target need to be expressed in the same cell type and (2) to better understand the molecular basis of human disease, it means that the tissues that specifically contribute to a disease need to be identified, isolated, and profiled. Subsequently, the targets of the known and novel regulatory RNAs need to be identified; this is currently a major challenge. It is known that miRNAs can bind dozens of targets and that single mRNAs can be regulated by multiple miRNAs. Canonical miRNA action is based on complementarity between the seed sequence of the miRNA and a binding sequence in the 3′-UTR of its target. This “rule” has been used to design algorithms that predict miRNA-binding sites in the 3′-UTRs of target mRNAs [see review by Hrdlickova et al. (18) for listing of web-based tools]. The field of lncRNA research is quite new and the functions of lncRNAs are still poorly understood. Although characteristics such as cell-type specificity and their role in epigenetic regulation also make lncRNAs interesting therapeutic targets, much more basic research is required to fully understand and appreciate the potential importance of lncRNAs in diagnostic work and treatment. There are currently no target prediction algorithms for lncRNAs, primarily because lncRNAs represent a heterogeneous group of regulatory RNAs that exhibit many different mechanisms of action. Moreover, their interaction with specific targets, which can be DNA or protein, appears to be determined by their structure rather than by their sequence. A factor that complicates lncRNA research is that they exhibit little sequence conservation throughout evolution. It is, therefore, not easy to identify mouse homologs of human lncRNAs for instance. However, it is possible that functional structures – independent of sequence – are more conserved, but there is not yet enough data available to prove definitely that this is a general feature. In contrast, miRNAs and their targets are well conserved throughout evolution, which makes it relatively easy to extrapolate mouse miRNA results to the human system.

Importantly, *in silico* predictions of targets and functions for regulatory RNAs need to be followed up by *in vitro* validation. Recently, advanced assays have been developed based on cross-linking of regulatory RNAs to their targets followed by analysis of their binding partners. By using such assays, miRNA targets can be identified by RNA sequencing [e.g., (179)] and lncRNA interacting partners can be determined by DNA sequencing or mass spectrometry [e.g., (180)]. These technically challenging assays are currently considered to be the state-of-the-art in pinpointing the targets of regulatory RNAs.

Much attention in the miRNA field is currently focused on the potential of using circulating miRNAs as biomarkers for disease. Tissue-specific expression profiles of miRNAs and lncRNAs are one of the features that make them attractive biomarker candidates. MiRNAs are of particular interest because it has been shown that they exhibit stability in plasma and serum, a feature that is not expected of lncRNAs. MiRNA (and lncRNA) levels can be measured by PCR, array technology, or by next generation sequencing applications, with each technique having its own advantages and disadvantages (181). Unfortunately, there are no simple tests (for instance dip-sticks) available to detect RNAs. Additionally, miRNAs are attractive targets for therapy. For instance, decoys can be applied to prevent miRNAs or lncRNAs from binding to their targets. Miravirsen, an inhibitor of miR-122, was shown to reduce HCV viremia in monkeys and is currently the most advanced miRNA-based drug in the clinical testing pipeline (Phase II). For more details on the potential of miRNAs or lncRNAs in the clinic and on the challenges associated with their delivery to target tissues, we recommend the reviews by Ling et al (182) and Li and Rana (183). It is clear, however, that the elucidation of more complete transcriptional networks, containing regulatory RNAs and protein-coding genes, will soon provide several more potential targets for therapeutic options.

## Conflict of Interest Statement

The authors declare that the research was conducted in the absence of any commercial or financial relationships that could be construed as a potential conflict of interest.

## Abbreviations

3′-UTR, 3′-untranslated region; Aicda, activation-induced cytidine deaminase; BACE1, gene encoding β-secretase-1; AGO, Argonaute; AP-1, activator protein-1; APAF1, apoptotic protease activating factor 1; APC, antigen-presenting cell; Bak1, Bcl-2 homologus antagonist/killer 1; BBB, blood–brain barrier; Bcl-2, B-cell lymphoma-2; BDNF, brain-derived neurothrophic factor; Bim, pro-apoptotic factor; BLIMP-1, B lymphocyte-induced maturation protein-1; BM, bone marrow; CaMKII, calcium/calmodulin-dependent protein kinase II; Cepba, CCAAT/enhancer binding protein-α; CCL1, chemokine (C-C motif) ligand 1; CD, Crohn’s disease; CDK, cyclin-dependent kinase; CeD, celiac disease; C-ETS, c-E26 transformation specific transcription factor; CLP, common lymphoid progenitor; CMP, common myeloid progenitor; CRC, colorectal carcinoma; CREB, cyclic AMP-responsive element binding protein; CSF, cerebrospinal fluid; CSF1, colony stimulating factor 1; CXCL10, C-X-C motif chemokine 10; DC, dendritic cell; DGCR8, DiGeorge syndrome critical region gene 8; DN, double negative; DOCK-1, dedicator of cytokinesis-1; DP, double positive (CD4+CD8+); DSS, dextran sulfate sodium; EAE, autoimmune encephalomyelitis; EBV, Epstein–Barr virus; eQTL, expression quantitative trait locus; FADD, Fas-associated death domain; Fo, follicular B-cell; Foxp3, forkhead box P3; GC, germinal center; GWAS, genome-wide association study; hnRNP, heterogeneous nuclear ribonucleoprotein; HOX, homeobox; HSC, hematopoietic stem cell; IBD, inflammatory bowel disease; IFN, interferon; IGF-1, insulin-like growth factor 1; Ikzf4, Ikaros family zinc finger 4; IL, interleukin; IPA, ingenuity pathway analysis; IRAK1, interleukin-1 receptor-associated kinase 1; IRF4, IFN regulatory factor 4; IRS-1, insulin regulatory subunit-1; iTreg, induced regulatory T-cell; JNK, JUN N-terminal kinase; KO, knockout; lincRNA, long intergenic ncRNA; LLRK2, leucine-rich repeat kinase-2; lncRNA, long non-coding RNA; LPS, lipopolysaccharide; Lt-α, lymphotoxin-α; MAPK, mitogen-activated protein kinase; MBP, oligodendrogial myelin basic protein; MEK, MAPK kinase; miRNA, micro-RNA; mirtron, intron-derived splicing-dependent miRNA; MS, multiple sclerosis; mTOR, mammalian target of rapamycin; MZ, marginal zone; MZF-1, myeloid zinc finger-1; NATs, natural antisense transcripts; ncRNA, non-coding RNA; NEAT1, nuclear enriched abundant transcript 1; NF-κB, nuclear factor-κB; NK, natural killer; PBMC, peripheral blood mononuclear cell; Phlpp2, PH domain and leucine-rich repeat protein phosphatase 2; PI3K, phosphoinositide 3-kinase; PP-MS, primary-progressive multiple sclerosis; PMN, polymorphonuclear cells; PRDM1, PR domain zinc finger protein 1; pre-miRNA, precursor-miRNA; pri-miRNA, primary miRNA; PTEN, phosphatase and tensin homolog; RISC, RNA-induced silencing complex; RR-MS, relapsing-remitting multiple sclerosis; RUNX-1, runt-related transcription factor 1; siRNA, small interference RNA; SFPQ, splicing factor proline/glutamine-rich; SOCS1, suppressor of cytokine signaling-1; simtron, intron-derived splicing-independent miRNA; sncRNA715, small non-coding RNA 715; SNP, single nucleotide polymorphism; SOD2, superoxide dismutase 2; SP-MS, secondary progressive multiple sclerosis; STAT, signal transducer and activator of transcription; T-bet, T-box expressed in T-cells; TCR, T-cell receptor; TGFβ, transforming growth factor β; TGFβR, TGFβ-receptor; Th, T helper; THRIL, TNFα- and hnRNPL-related immunoregulatory lincRNA; TLR, Toll-like receptor; TNF, tumor necrosis factor; TRAF, TNF receptor-associated factor; Treg, regulatory T-cell; UC, ulcerative colitis; VE-cadherin, vascular endothelial-cadherin; XPO5, exportin 5.
